# The Evaluation of Serum Ascites Albumin Gradient and Portal Hypertensive changes in Cirrhotic Patients with Ascites

**DOI:** 10.5005/jp-journals-10018-1157

**Published:** 2016-07-09

**Authors:** Forhad Hossain Md Shahed, Mamun-Al-Mahtab, Salimur Rahman

**Affiliations:** 1 Department of Medicine, Sadar Hospital, Brahmanbaria, Bangladesh; 2 Department of Hepatology, Bangabandhu Sheikh Mujib Medical University, Shahbagh, Dhaka, Bangladesh

**Keywords:** Cirrhosis, Esophageal varices, Gastric varix, Portal hypertension gastropathy, Serum ascites albumin gradient.

## Abstract

**Introduction:**

Ascites is a common complication of chronic liver diseases and is related to the extent of portal hypertension. This study evaluated whether the serum ascites albumin gradient (SAAG) (the difference between the albumin level of serum and of ascitic fluid) is endowed with clinical implications.

**Materials and methods:**

This is a prospective study involving 50 patients with cirrhosis of liver with ascites. The SAAG was measured in all patients and its relation with portal hypertensive changes was analyzed.

**Results:**

Based on SAAG values, the patients were divided into three groups: Group 1 – SAAG value 1.1 to 1.49 gm/dL (n = 15); group 2 – SAAG value 1.5 to 1.99 gm/dL (n = 9); and group 3 – SAAG value 2.0 gm/dL (n = 26). In group 1, 14 patients had esophageal varices (93.3%) and 13 had gastropathy (86.6%). In group 2, all 9 patients had esophageal varices (100%), 7 (77.7%) had gastropathy, and 1 (11.1%) had gastric varices. In group 3, all 26 patients had esophageal varices (100%), 24 patients (92.3%) had gastropathy, and 1 patients (3.8%) had gastric varices.

**Conclusion:**

Serum ascites albumin gradient value is weakly related to the extent of portal hypertension in patients with liver cirrhosis and its implication seems to be limited in clinics.

**How to cite this article:**

Shahed FHM, Al-Mahtab M, Rahman S. The Evaluation of Serum Ascites Albumin Gradient and Portal Hypertensive changes in Cirrhotic Patients with Ascites. Euroasian J Hepato-Gastroenterol 2016;6(1):8-9.

## INTRODUCTION

Ascites, defined as a pathological accumulation of fluid in peritoneal cavity, may accompany several diseases.^1^ Chronic liver disease, especially cirrhosis, is related with ascites. The serum ascites albumin gradient (SAAG), which is based on the difference between the albumin level of serum and of ascitic fluid, may be used to assess the extent of ascites.^2^ The SAAG is thought to reflect the colloid osmotic pressure gradient and the degree of portal hypertension. It has been shown that SAAG is a better discriminator of portal hypertension than ascites. Patients with SAAG ≥ 1.1 gm/dL is considered as having high SAAG, indicating the presence of portal hypertension, while those with SAAG < 1.1 gm/dL are considered as having low SAAG, indicating the absence of portal hypertension. Portal hypertensive changes in upper gastrointestinal as noted by endoscopy are esophageal varices, gastric varices, and portal hypertensive gastropathy.^3^ The SAAG is proposed to be a factor determining the degree of portal hypertension and prognosis of patients with cirrhosis. We assessed the relation between SAAG and portal hypertensive changes in this study.

## MATERIALS AND METHODS

This is a prospective study involving 50 patients with liver cirrhosis with ascites. This study was conducted in the Department of Hepatology, Bangabandhu Sheikh Mujib Medical University, Dhaka, Bangladesh, from January to December 2005. The age range of the patients was 15 to 70 years. Both male and female patients were included in the study. Patients of cirrhosis with ascites with high SAAG values (≥ 1.1 gm/dL) were enrolled. The following patients were excluded from the study: Cirrhotic patients with ascites with low SAAG values (< 1.1 gm/dL), pregnant women, patients with space-occupying lesion in the liver, patients with intra abdominal tuberculosis or malignancy, patients who had undergone endoscopic treatment for esophageal varices previously, and patients in whom endoscopy was contraindicated. Biochemical liver function tests and viral serological markers were done. Ultrasonography of the hepatobiliary system was done to detect the presence of features of chronic liver diseases and of ascites and to exclude space-occupying lesion in the liver. Ascitic fluid was aspirated from the abdominal wall. The ascitic fluid was sent for cytology and assessment of total protein, albumin, and malignant cell. At the same time, venous blood was drawn and sent for serum albumin concentration estimation. After obtaining reports, SAAG values were determined. Endoscopy of the upper gastrointestinal tract was done by Olympus video endoscopy. If esophageal varices were present, then the number of esophageal varices, the size of esophageal varices, and the presence of any red signs over the varices were noted. The stomach was checked for the presence of portal hypertensive gastropathy and gastric varix. Detailed history of each patient was taken. All data were analyzed in a personal computer by Statistical Package for the Social Sciences (SPSS) program. Significance of the test was tested by chi-square test. The p value of < 0.05 was taken as statistically significant. Correlation analysis was done by pearson correlation test.

## RESULTS

The prospective study was done to assess if SAAG value can be used as a preliminary indicator of the presence of portal hypertensive changes and also to assess the correlation between SAAG values and portal hypertensive changes in cirrhotic patients with ascites. The SAAG values and conditions of esophageal varices for the three groups of patients have been in Table 1.

**Table 1:** **Table 1:** Serum ascites albumin gradient values and extent of esophageal varices

				*Size of esophageal varices no. of patient (%)*											
*SAAG group (gm/dL)*		*No. of patient*		*Absent*		*Small*		*Medium*		*Large*		*r*		*p-value*	
1.1–1.49		15		1 (6.66%)		8 (53.28%)		4 (26.64)		2 (13.32%)		0.358		0.011	
1.5–1.99		9		0		1 (11.11%)		6 (66.66%)		2 (22.22%)					
≥ 2		26		0		9 (34.2%)		5 (19.0%)		12 (45.6%)					
Total (%)		50 (100%)		1 (2.0%)		18 (36.0%)		15 (30.0%)		16 (32.0%)					

In patients with SAAG 1.1–1.49 gm/dL, 14 of 15 patients (93.3%) had esophageal varices and 13 of 15 patients (86.6%) had gastropathy. In patients with SAAG 1.5–1.99 gm/dL, 9 of 9 patients (100%) had esophageal varices and 7 of 9 patients (77.7%) had gastropathy. In SAAG ≥ 2 gm/dL, 26 of 26 patients (100%) had esophageal varices and 24 of 26 patients (92.3%) had gastropathy.

## DISCUSSION

The SAAG is a physiological clinical diagnostic tool for the evaluation of ascites. An increased SAAG (> 1.1 gm/dL) value indicates the presence of portal hypertension, which is detected by observing portal hypertensive changes in the upper gastrointestinal tract.^4^ Several studies about SAAG have been conducted in different parts of the world with regard to alcoholic cirrhosis, but studies on nonalcoholic cirrhosis are scanty. In our study, a total 50 patients of cirrhosis of various etiologies were included. A relation was studied between SAAG and portal hypertensive changes in the upper gastrointestinal tract like esophageal varices, gastric varices, and gastropathy. As the SAAG value rises, the percentage of patients having large-sized varices increases, but such increase was not seen in small- and medium-sized varices. In patients with SAAG 1.1 to 1.49 gm/dL, 13 of 15 had mild-grade and 5 (33.33%) had severe-grade Portal hypertensive gastropathy (PHG). In patients with SAAG 1.5 to 1.99 gm/dL, 7 of 9 (77.77%) had PHG, of which 4 (44.44%) had mild-grade and 3 (33.33%) had severe-grade PHG. In patients with SAAG 2 gm/dL, 24 of 26 (92.31%) had PHG, of which 11 (42.30%) had mild-grade and 13 (49.99%) had severe-grade PHG. As the SAAG value rises, the percentage of patients with severe-grade also rises, but such increase was not seen in mild cases. Gastric varices were present in only 2 of 50 patients: 1 of 9 patients (11.11%) in SAAG group 1.5 to 1.99 gm/dL and 1 of 26 patients (3.84%) in SAAG group 2 gm/dL (p > 0.05). No, child grade means grade of severity of cirrhosis according to scientist name child pugh . Taken together, it seems that SAAG assessment may not have significant implication in a clinical setup in Bangladesh.
